# Recent Insights and Novel Bioinformatics Tools to Understand the Role of MicroRNAs Binding to 5′ Untranslated Region

**DOI:** 10.3390/ijms14010480

**Published:** 2012-12-27

**Authors:** Letizia Da Sacco, Andrea Masotti

**Affiliations:** Gene Expression—Microarrays Laboratory, Bambino Gesù Children’s Hospital, IRCCS, P.za S. Onofrio 4, Rome 00165, Italy; E-Mail: letizia.dasacco@opbg.net

**Keywords:** miRNAs, 5′UTR, 3′UTR, bioinformatics tools, translation regulation

## Abstract

MicroRNAs (miRNAs) are small non-coding RNAs that regulate gene expression through the binding of the 3′ untranslated region (3′UTR) of specific mRNAs. MiRNAs are post-transcriptional regulators and determine the repression of translation processes or the degradation of mRNA targets. Recently, another kind of miRNA-mediated regulation of translation (repression or activation) involving the binding of miRNA to the 5′UTR of target gene has been reported. The possible interactions and the mechanism of action have been reported in many works that we reviewed here. Moreover, we discussed also the available bioinformatics tools for predicting the miRNA binding sites in the 5′UTR and public databases collecting this information.

## 1. Introduction

MicroRNAs (miRNAs) are ~22-nt small non-coding RNAs that regulate the gene expression post-transcriptionally through perfect or imperfect base pairing with 3′ untranslated region (3′UTR) of their target mRNAs [1]. Generally, miRNAs are expressed in a tissue-specific manner and in particular developmental stages [2–4]. Besides, miRNAs are deregulated in several human diseases [5]. Up to now, the most studied role of miRNAs is as a repressor of mRNA levels and translation [6].

Recent studies have outlined that animal miRNAs are able not only to repress, but also activate, gene expression, acting on mRNA stability and translation regulation [6,7]. Although this mechanism has still to be completely elucidated, some recent studies started to investigate different aspects of miRNA-dependent activation of translation.

In this review, we discussed the studies where miRNAs have been reported to regulate gene expression through their binding to the 5′UTR of target genes [7]. This novel mechanism of action has been studied by using *in vitro*, *in vivo* and *in silico* approaches. From a bioinformatics point of view, a great number of new databases and tools have been developed to predict miRNAs target sites in 5′UTR of mRNA targets and to unravel their functional role, and this review is aimed at discussing these topics.

## 2. The Translation Process

The translation process can be generally divided into three phases: initiation, elongation and termination [8]. Every single stage involves many protein factors. The most complex phase is represented by the initiation process, which is also the rate-limiting step. In fact, in order for translation to take place, the majority of eukariote mRNAs require a 5′ end of 7-methylguanosine (the m^7^G cap), although few mRNAs [9] and many viral RNAs are translated in a cap-independent manner [10].

**Figure 1:**
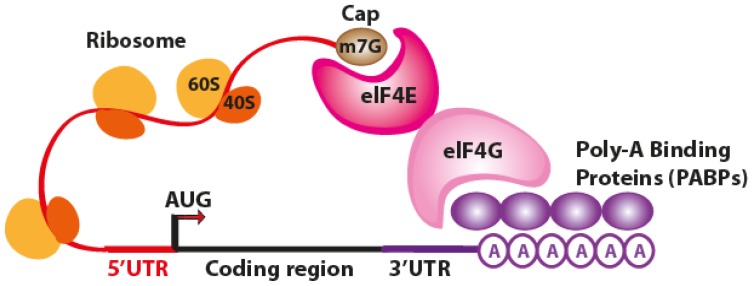
Schematic representation of cap-dependent translation. The initiation factor eIF4E recognizes the 5′cap and promotes the recruitment of eIF4G generating the initiation complex. Ribosome is recruited at the 5′ end of mRNA, and translation takes place once the scanning of 5′UTR is complete and the AUG codon is reached. (Adapted from [11]).

In the cap-dependent translation, the eukaryotic initiation factor 4E (eIF4E) recognizes the 5′cap and promotes the recruitment of other initiation factors (*i.e.*, eIF4G) for the assembly of the initiation complex. Moreover, eIF4G binds also some poly(A)-binding proteins (PABPs), leading to the formation of a “closed” mRNA loop that further enhances ribosome recruitment. When ribosome is recruited to the 5′ end of mRNA, translation takes place once the start AUG codon is reached after 5′ untranslated region (5′UTR) scanning (Figure 1).

In the cap-independent translation, the ribosome binds directly to an internal ribosome entry site (IRES), a nucleotide sequence within a given mRNA sequence and located near the start codon [12]. For example, IRES sequences in hepatitis C virus (HCV) allow it to bypass the recruitment of initiation factor proteins of the eIF4 family and to start the translation [13].

## 3. Post-Transcriptional Gene Regulation by miRNAs Binding to the 3′UTR of Target Genes

It is generally acknowledged that a single miRNA can deregulate the expression levels of many target genes by a post-transcriptional mechanism [14]. The miRNA-dependent gene expression regulation is mediated by the RNA-induced silencing complex (RISC) containing Dicer and many other associated proteins [15]. RISC incorporates only one functional miRNA strand, and it is often called “miRISC”. Members of the Argonaute (Ago) protein family are central to the RISC function. miRNAs form stable complexes with Argonaute proteins (e.g., AGO2), which represent the core of the silencing complex. However, the binding of miRNAs to mRNA targets can either repress or activate the translation process.

### 3.1. Translation Repression

**Figure 2:**
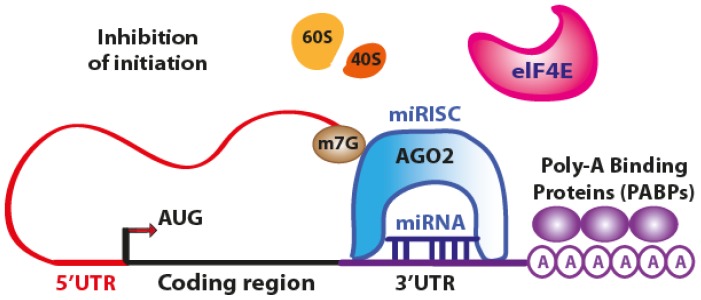
Schematic representation of miRNA-mediated inhibition of translation. The RISC complex loaded with a miRNA interferes with the cap recognition by eIF4F and the recruitment of ribosomal subunits 60S and 40S, preventing the ribosome complex formation and leading to inhibition of translation initiation. (Adapted from [11]).

The initiation of translation can be inhibited owing to the fact that AGO2 protein binds both the m^7^G cap and the 3′UTR of target mRNA at the same time, therefore preventing the recognition of the cap by eIF4E and the access to mRNA by the translation apparatus (Figure 2).

**Figure 3:**
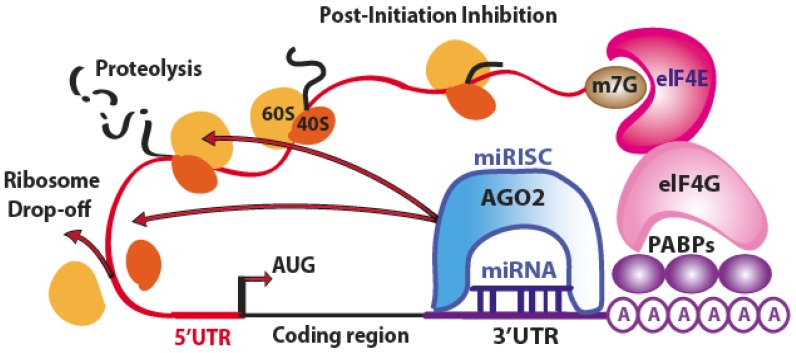
Schematic representation of miRNA-mediated post-initiation inhibition. Among the various mechanistic hypotheses, the miRISC complex may inhibit the ribosomal elongation, induce ribosome drop-off or facilitate the degradation of nascent polypeptides. (Adapted from [11]).

However, many authors observed that miRNAs can affect the translation process not only by inhibiting the translation initiation process, but also intervening at post-initiation level [7,8]. At this level, an additional mechanism of ribosome drop-off is also possible [16]. This process consists in a premature termination of translation and degradation of the incomplete forming protein (Figure 3).

**Figure 4:**
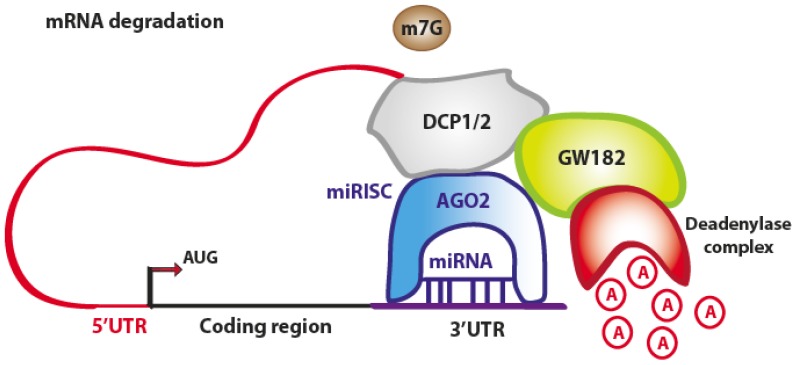
Schematic representation of miRNA-mediated mRNA degradation. The AGO2 protein interacts with GW182, whereas the enzymes DCP1/2 remove the 5′cap. Exonucleases start the deadenylation process, leading to the degradation of the mRNA. (Adapted from [11]).

The miRNA-mediated mRNA degradation is the result of mRNA terminal deadenylation by 3′→5′ exoribonucleases, 5′-terminal cap removal by decapping enzymes DCP1/2 and hydrolysis of the remaining portion of mRNA by 5′→3′ exonucleases [17]. The DCP1/2 enzymes require the interaction with the RISC components AGO2 and GW182, whereas the interaction between PABPs and GW182 enhances the mRNA deadenylation process, although PABPs are not directly responsible for it (Figure 4) [18].

The mRNA translational repression is a process localized to eukaryotic mRNA processing bodies (P bodies) and related ribonucleoprotein (RNP) granules, known as stress granules [19]. These complexes contain proteins, such as the initiation factor eIF4E and other decay factors, but they lack of ribosomes. Interestingly, P-bodies are temporary storage sites for repressed mRNAs, which are not necessarily destined to degradation. In fact, following general reactivation of cellular protein synthesis or in response to environmental stimuli, mRNAs can exit P-bodies and return to be available for the translation process [20].

### 3.2. Translation Activation

Recently, some papers outlined that miRNAs and their associated protein complexes (microRNPs) can enhance the gene expression at the post-transcriptional level [7,21,22]. The authors found that the observed upregulation can be mediated (activated) either directly by distinct microRNPs or indirectly as a result of the regulatory effects on microRNA-mediated repression (relief of repression). In particular, the authors identified a novel microRNP complex acting as a key player for the translation activation in *Xenopus laevis* immature oocytes and also in mammalian cells [21]. They also reported that miRNA-mediated upregulation of target mRNAs in oocytes is dependent on nuclear entry of the miRNA, whereas cytoplasmically-injected miRNA represses target mRNAs. The nuclear microRNP is formed by components, such as AGO2, a specific isoform of the Fragile-x-mental retardation related protein 1 (FXR1) family of proteins and miRNAs (*i.e.*, miR-16). The importance of these studies rely on having emphasized that the compartmentalization of AGO2-FXR1-iso-a complex is necessary for selective recruitment of specific mRNAs and the following miRNA-mediated upregulation. Moreover, this partially explored mechanism can be only a part of a more complex regulatory process, and many other protein complexes may exist. Once more, the exact role of miRNAs binding to 3′UTR sequences will surely ensure further interesting cues.

## 4. Post-Transcriptional Gene Regulation by miRNAs Binding to the 5′UTR of Target Genes

Lytle and colleagues experimentally demonstrated that miRNAs can bind efficiently to any position of a given mRNA target (5′ or 3′UTRs) and repress the translation at some step downstream of initiation [23].

MiRNAs targeting 5′UTR have been also quite recently studied in *in vitro* experiments using specific gene reporter assays [23,24] and with bioinformatics approaches [24,25].

The binding of miRNAs to the 5′UTR of target genes has been reported to repress or activate translation. We summarized these studies in Table 1, and we discussed them according to miRNAs functioning as repressors or activators.

### 4.1. Translation Repression

Translation is a process that can be finely regulated by miRNAs. In fact, miRNAs can bind not only the 3′UTR, but also the 5′UTR (or both) of target mRNA. While the binding with 3′UTR has been thoroughly investigated in the last decade, the binding mechanism to 5′UTR and the specificity of such a regulation is still not completely understood.

The first example of miRNA-mediated translation regulation by 5′UTR binding has been reported for *Drosophila* miR-2 [26]. The authors systematically investigated how the position of miRNA binding sites influences translational regulation, showing that translational regulation is elicited *in vitro* and *in vivo* not only from the 3′UTR, but also from six miR-2 binding sites located in the 5′UTR or in the ORF. They also demonstrated that miR-2 triggers mRNA deadenylation and inhibits translation initiation in a cap-dependent manner. They also observed deadenylation processes, the formation of pseudopolysomes and a reduction of 80S complex formation, indicative of a miR-2-mediated block of translation initiation. Interestingly, they observed that single or dual miRNA binding sites in the 5′UTR or the ORF are generally less functional than in the 3′UTR or even completely not functional. These *in vitro* studies have been also validated in *Drosophila* embryo extracts. This work represents the first demonstration that miRNA binding sites in the 5′UTR or in the ORF can influence translation similarly to what is observed for miRNA binding 3′UTR sites.

Another example of miRNA regulatory module consisting of the proto-oncogene FOS-like antigen-1 (FOSL1) and the candidate tumor suppressor miR-138 has been recently reported [27]. The authors confirmed the miR-138-mediated downregulation of FOSL1 in squamous cell carcinoma cell lines and demonstrated the contribution of this miRNA to tumorigenesis. From bioinformatics predictions, they found that miR-138 binds to three canonical and three high affinity non-canonical target sites of the FOSL1 gene: one in the 5′UTR, three overlapping sites in the coding sequence (CDS), and two overlapping sites in the 3′UTR. By ribonucleoprotein-immunoprecipitation assays, the authors demonstrated the miR-138-directed recruitment of FOSL1 mRNA to the RISC complex, which led to its downregulation. The proto-oncogene FOSL1 dimerizes with proteins of the Jun family, resulting in the formation of the Activator Protein 1 (AP-1) complex. This transcription factor is crucial for many processes, as it controls differentiation, proliferation and apoptosis. The miR-138-mediated decrease of FOSL1 protein level also determines a down-regulation of the transcription repressor gene Snail homolog 2 (SNAI2) and, as a direct consequence, an enhanced E-cadherin expression, which prevents epithelial-to-mesenchymal transition and cancer progression.

### 4.2. Translation Activation

Although the majority of predicted and experimentally validated miRNA sites are located in the 3′UTR of a given mRNA, animal miRNAs may also target 5′UTR and coding regions, according to experiments involving both artificial and natural mRNAs and also by bioinformatics predictions [36,38,39]. It has been observed that the association of miRNAs with 5′UTR generally induces an activation of translation rather than a repression [28,30,31].

**Figure 5:**
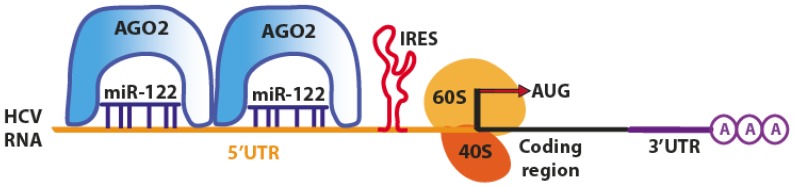
Liver specific miR-122 stimulates translation of HCV RNA through direct binding to two target sites in the 5′-UTR. (Adapted from [7]).

One of the first examples of miRNA-dependent activation of translation by 5′UTR binding is the Hepatitis C virus (HCV), which evolutionarily developed a mechanism to exploit the miRNA machinery for its replication. HCV employs the host liver-specific miR-122 as a positive regulator of viral replication, leading to the accumulation of the RNA viral genome [28,40]. In mammals, the liver-specific miR-122 is involved in regulating lipid and cholesterol metabolism [41]. It has been observed that HCV RNA can replicate in hepatocyte-derived cellular carcinoma (HuH-7) cells, which express miR-122, but not in HepG2 cells, which do not express miR-122 [28]. Moreover, silencing of miR-122 in HuH-7 cells resulted in a marked loss of replicating viral RNA, suggesting that miR-122 sustains the existence of HCV in the liver (hepatotropic virus). The HCV genome has three sites of binding for miR-122: the first is located in the 3′UTR, whereas the other two are in the 5′UTR, upstream of the HCV IRES, responsible for virus translation (Figure 5).

The miRNA binding to 5′UTR does not affect the RNA stability, but enhances translation, ultimately increasing the abundance of HCV RNA [32,33,40]. Another study reported that miR-122 favored the association of ribosomal initiation complexes, increasing the formation of complete 80s ribosomes [30,31]. Jangra and colleagues observed the same translation enhancement, and they proposed a mechanism where miR-122 induces a conformational change of HCV IRES (in the 5′UTR) by modifying the structure from an inactive (closed) conformation, to a more active (open) conformation, finally promoting translation [31]. However, it has been recently demonstrated that translation activation does not involve a structural transition in the HCV IRES and that the process is mediated by Argonaute proteins [34]. Ago2 and miR-122 act cooperatively to protect the viral genome from 5′ exonuclease activity of the host mRNA decay machinery. Thus, miR-122 acts in an unconventional way by stabilizing HCV RNA and slowing its decay [35].

**Figure 6:**
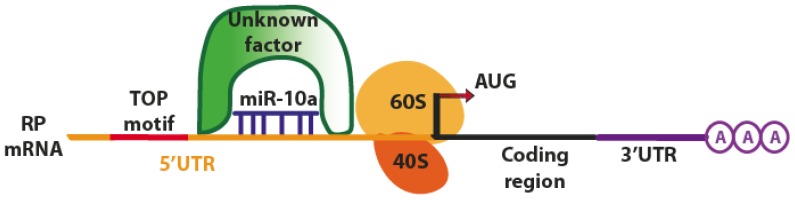
The binding site of miR-10a has been identified downstream of the regulatory 5′ oligopyrimidine tract (5′TOP) motif of ribosomal proteins (RP) mRNAs, although with an incomplete base pairing between the 5′UTR and the miRNA seed region.

Another example of active regulation by miRNAs has been reported by Orom and collaborators, who demonstrated that miR-10a interacts with the 5′UTR of mRNA encoding ribosomal proteins (RP), resulting in their translational enhancement [36]. The binding site of miR-10a has been identified downstream of the regulatory 5′ oligopyrimidine tract (5′TOP) motif of RP mRNAs, although with an incomplete base pairing between the 5′UTR and the miRNA seed region. The transfection with exogenous miR-10a significantly increased the amount of newly synthesized RPs and led to a 30% increase of protein expression, suggesting that mir-10a has a role in controlling ribosome biogenesis and protein synthesis. Moreover, the authors demonstrated that miR-10a can alleviate the translational repression induced upon amino acid starvation. Besides, 5′TOP motifs are also involved in sensitivity to nutrients (Figure 6) [42].

The exact mechanism of miRNA regulation via 5′TOP motif is still not completely clear and has to be further investigated. The authors suggested that miR-10a might compete with the binding of inhibitor factors downstream of the 5′TOP motif. However, this factor has never been identified.

Another enhancing effect of a specific miRNA that targets 5′UTR has been also reported for the receptor-interacting protein 140 (RIP140), a transcriptional corepressor that regulates diverse genes, such as those responsive to hormones and involved in metabolic processes [37]. The authors identified a novel 5′ splice variant of RIP140 mRNA in mouse brain and P19 embryonal carcinoma cells and identified a target sequence for miR-346 in the 5′UTR of RIP140 mRNA. Moreover, they found that miR-346 elevates RIP140 protein levels by facilitating association of mRNA with the polysome fraction without affecting mRNA stability. Also, in this case, further studies are needed to unravel the role of miRNA binding to 5′UTR site.

## 5. Bioinformatics

In the previous paragraphs, we have briefly reviewed the experimental evidences of miRNAs binding to the 3′UTR and the 5′UTR of target genes. However, the mechanisms of interaction at 5′UTR and the mode of action have still to be thoroughly investigated. To help unravel the overall scenario of miRNA-mediated translational regulation, novel bioinformatics tools have been developed, but many more have to be designed in order to predict miRNA binding sites in the 5′UTR and in the coding sequence (CDS) of mRNA targets. In fact, most common computational programs, such as miRanda [43], DIANA-microT [44], RNAhybrid [45], TargetScan [46], MicroInspector [47], PicTar [48], miTarget [49], RNA22 [50], PITA [51] and microTar [52] have been developed to predict miRNA target sites, mainly at the 3′UTR of target genes. These algorithms generally rely on two distinct features when performing predictions: (1) the complementarity between the seed sequence at the miRNA 5′ end and (2) an extensive base pairing to the 3′ end of the miRNA to compensate for imperfect or a short stretch of base pairing to the seed portion of the miRNA [53].

In recent years, several bioinformatics tools have been developed to study the interactions between miRNAs and 5′UTR (or CDS), which have been summarized in Table 2.

### 5.1. miBridge

Lee and colleagues were the first to identify a new class of targets containing both 5′UTR and 3′UTR sites for miRNA binding, with the 5′UTR sites predicted to interact with the 3′ end of mature miRNAs [24]. Exploiting hybridization energy and sequence matches in their algorithm, they identified a huge number of endogenous motifs in the 5′UTRs, complementary to the 3′ end of miRNAs. To reduce complexity, they predicted also the target sites in the 3′UTR using the canonical binding from the 5′ end of miRNA, resulting in a “combined” 5′UTR–3′UTR binding search strategy that they called ‘5UTR:3emiR’ (miRNA recognition site in the 5′UTR and the binding with the 3′ end for a given miRNA). Owing to the peculiar characteristics of miRNAs binding both 5′UTR and 3′UTR (resembling a bridge), they called their bioinformatics tools “miBridge”. To validate the *in silico* target predictions obtained with miBridge, the authors assessed the gene expression level of AXIN2 (target of miR-34a) and SEC24D (target of miR-605), finding them translationally repressed (Table 1). These two genes have been predicted not only by the commonest miRNA target prediction algorithms (such as TargetScan), but also by miBridge. By focusing on those mRNA targets regulated by miRNAs at both 5′ and 3′ UTRs, the authors suggested that their approach can help to avoid false positive predictions and restrict the number of miRNA targeted target genes.

Zhou and collaborators predicted miRNA target sites in the 5′UTR, CDS and 3′UTR of *Homo sapiens*, *Mus musculus* and *Drosophila melanogaster* using the two most common prediction tools miRanda and TargetScan [25]. Interestingly, they found that the 5′ UTR has more putative target sites than the 3′ UTR after normalization with the average length of the respective region. Moreover, they found that putative target sites were more conserved than non-target regions in both the 5′UTR and 3′UTR, implying that binding sites in the 5′UTR are subject to high selective pressure and might be functional. The authors performed also various experimental validation employing both artificial and natural mRNAs, demonstrating a functional role of miRNA target sites in the 5′UTR and CDS. It is noteworthy this study emphasize once more that the understanding of this mechanism could dramatically improve the accuracy of target-site prediction algorithms. Unfortunately, the authors have not implemented a searchable on-line database, and the pre-compiled predictions have been reported only as supplementary data along with the manuscript.

### 5.2. miRTar

The integrated resource miRTar has been developed for the identification of miRNA target sites by using a combination of TargetScan, miRanda, PITA and RNAHybrid algorithms [54]. MiRTar generates the potential miRNA-target gene interaction pathway and supports three major features. The first one is to consider seven different scenarios (single miRNA to single gene, single miRNA to multiple genes, multiple miRNAs to single gene, multiple miRNAs to multiple genes, all miRNAs to multiple genes and multiple miRNAs to metabolic pathways) to identify the regulatory relationships between miRNAs and their targets (binding to 5′UTR, 3′UTR or coding region). The second feature consists in a gene set enrichment analysis for predicted miRNA-regulated genes to classify them in KEGG pathways and elucidate their biological role. The last feature covered by miRTar consists in providing a viewpoint on the regulation between miRNA and RNA alternative splicing. In fact, miRNA target sites located in alternatively spliced exons of a specific gene can present a potential regulatory role, since the target site can be conditionally spliced out and cannot be included in the gene transcript. Therefore, RNA alternative splicing induced by miRNAs can cause incomplete gene suppression and affect miRNA regulations in diverse protein functions. Therefore, miRTar is a tool that allows biologists to identify the biological functions and regulatory relationships between protein coding genes and miRNAs in a straightforward and intuitive way. The main advantage is the clear presentation of the various web sections and the possibility to download various datasets (miRNA interactions with 5′UTR, 3′UTR, CDS or any region). Unfortunately, the tool is limited only to human predictions.

### 5.3. miRWalk

Another recent repository is miRWalk, which is a comprehensive database providing miRNA binding sites, already validated or predicted not only in the 3′ UTR, but also in the promoter region, as well as in the 5′UTR and in the CDS of all known and mitochondrial genes (literally a “walking”) in humans, mice and rats [55]. Therefore, the miRWalk web interface has been divided into two modules (predicted and validated targets). The predicted target module is classified into six parts: Target Gene, miRNA, Pathway, Chromosome, OMIM and Mitochondrial Target. The Validated Target module has different search pages, but the organization is similar: Target Gene, miRNA, Pathway, Disease, Organ, Cell line, miRNA literature, OMIM disorder and miRNA Processing Proteins. The miRWalk overall architecture provides a more holistic view of genetic networks of miRNA-gene-pathways and miRNA-gene-OMIM disorder interactions and integrates biological data with literature information. Besides, the miRWalk database hosts 98,887 relationships on 1572 miRNAs from human, mouse and rat linked to 691 diseases, thus representing an important resource for gathering information on miRNAs linked to human diseases. This tool has the advantage of easily getingt an overall picture of the biological system under investigation, but also, in this case, it is limited only to humans.

### 5.4. Sfold-STarMirDB

Another web resource and database for statistical folding of nucleic acids and studies of regulatory RNAs (named Sfold) has been developed by Ding and colleagues [56]. The Sfold web site contains various modules for different predictions and calculations (*i.e.*, target accessibility prediction and RNA duplex thermodynamics for siRNA, oligonucleotides and probe design, statistical RNA folding, energetic calculations between structured target and miRNAs) and a link for a database of microRNA binding sites predicted by STarMir [55]. STarMir can be interrogated by human or mouse model (V-CLIP- and HITS-CLIP-based models) and by specifying the single miRNA and the target sequences. STarMirDB integrates a search module (by specifying miRNA names and gene accession number) and a compiled collection of features and predictions for 3′UTR-seed or 3′UTR-seedles sites, and the correspondent prediction for 5′UTRs and CDS. This resource, although limited only to a small number of miRNAs considered, is, however, a useful resource that allows the performance of thermodynamic calculations and the derivation of energetic information on miRNA/target interactions [57].

### 5.5. miRNA_Targets

The most recent repository of genome-wide full-length mRNA/miRNA target prediction tools is miRNA_Targets [58]. This database has versatile search capabilities and visualization tools and contains target predictions for miRNA’s on 5′ UTRs, coding region and 3′ UTRs of all mRNAs. The predictions have been calculated for human, mouse, cow, chicken, zebrafish, fruitfly and *Caenorhabditis elegans* using two different target prediction algorithms, miRanda and RNAhybrid. The web version of the database allows also the analysis of miRNA target genes in a given set of down regulated genes and to compare gene ontology of two sets of miRNA target genes. Unfortunately, there is not a dedicated section for downloading the whole dataset of predictions, and there is not the possibility to integrate the separate predictions obtained with the two available algorithms.

## 6. Conclusions

From the studies and bioinformatics tools discussed in this review, it has been clearly outlined that novel molecular functions and mechanisms of action are continuously emerging for miRNAs. Interestingly, the classic binding mode (through the seed region typically encompassing the 5′ bases 2–7 of the miRNA) to the 3′UTR has been shown not to be the unique existing binding mode. In fact, also, the 5′UTR region can be targeted by a given miRNA, and evidence is emerging that the CDS can also play a role in post-transcriptional gene expression regulation and mRNA translation activity.

Of note, the work of Lee and collaborators [24] clearly suggested that novel binding modes (through the 3′ end of the miRNA) are also possible. These novel binding mechanisms inevitably contribute to a depiction of a more intricate picture of miRNA-mediated gene expression regulation than ever imagined before. Inevitably, these evidences suggest that the structural aspects of miRNA/mRNA binding should also be considered in further studies. In fact, other studies by Zhang and colleagues pointed out for the first time that the folding structure of other non-coding RNAs, such as long non-coding RNAs (lncRNAs), RNA molecules very close to miRNAs, is important to impart peculiar biological functions, as well as their primary sequences [59,60]. Therefore, we can imagine that structural aspects related to miRNA/mRNA interactions could also be crucial factors determining different outcomes and biological functions. We recently reviewed the challenges connected to the lncRNAs functional analysis and the available bioinformatics tools available to researchers interested in exploring this interesting field [61].

One of the crucial points that should be addressed in future studies is still the identification of “true” miRNA targets from the huge amount of predictions obtained by different algorithms. The 5′UTR binding could be used as a winning “filtering” strategy, once the method is confirmed on a greater number of experimentally validated targets.

Curiously, the advent of high-throughput technologies, such as next generation sequencing, has shifted the attention to novel RNA species (*i.e.*, long non-coding RNAs, nuclear and nucleolar RNAs) and their role in epigenetics. Little attention has been paid to alternative miRNA binding modes, and 5′UTR binding is only one example. We hope that our overview of alternative binding modes can stimulate the interest not only of biologists, but also of bioinformaticians for the development of novel tools able to fill the knowledge gap in miRNA-mediated gene expression and translation regulation.

## Figures and Tables

**Table 1:** Experimental studies involving miRNAs binding to 5′UTR. Target genes, mechanism of translational regulation and the biological process in which they are involved are also reported.

miRNA Name	Target mRNA or protein	Function/Process/Interaction	Reference
**Translation repression**			
miR-2 (Drosophila)	luciferase reporter gene	Deadenylation and formation of pseudopolysomes and reduction of 80S complex formation	[26]
miR-138 (Human)	FOSL1	Tumor suppressor microRNA. Down regulation of FOS-like antigen-1 (FOSL1) affects snail homolog 2 (SNAI2) and repression of E-cadherin. Contribution to tumorigenesis, cancer initiation and progression	[27]
miR-34a (Human)	AXIN2 luciferase reporter assay	miBridge interaction (miRNA binding both 5′ and 3′ UTRs)	[24]
miR-605 (Human)	SEC24D luciferase reporter assay	miBridge interaction (miRNA binding both 5′ and 3′ UTRs)	[24]
**Translation activation**			
miR-122 (Human)	HCV viral genome	Activation of translation of HCV mRNA, increase of 48s association in the initiation complex at the 5′UTR of viral genome. Accumulation of viral RNA and more efficient replication of HCV.	[28–35]
miR-10a (Human)	mRNA Ribosomal Proteins	Binding to 5′ TOP of mRNAs stimulating translation and decreasing the repression induced by amino acid starvation. Control of ribosome biogenesis and global protein synthesis, oncogenic potential in transforming cells. 5′TOP regulation of cellular stress response.	[36]
miR-346 (Human)	RIP140	Increase of the RIP140 level and direct increase of its repression activity. MiRNA is involved in the regulatory network to maintain the homeostasis in hormonal responses and metabolism.	[37]

**Table 2:** Bioinformatics resources (database and prediction tools) for studying the miRNA binding at 5′UTR.

Bioinformatics Resources	Year	Description	Web Link	Reference
miBridge	2009	Algorithm and Database	http://sitemaker.umich.edu/mibridge/home	[24]
miRTar	2011	Bioinformatics Tools integrated with KEGG pathways	http://miRTar.mbc.nctu.edu.tw	[54]
miRWalk	2011	Searchable database	http://mirwalk.uni-hd.de	[55]
Sfold-STarMirDB	2007	Algorithm and Database	http://sfold.wadsworth.org/cgi-bin/index.pl http://sfold.wadsworth.org/starmirDB.php	[56,57]
MiRNA_Targets	2012	Database and GO classification	http://mamsap.it.deakin.edu.au/mirna_targets/	[58]
